# National Trends in Main Causes of Hospitalization: A Multi-Cohort Register Study of the Finnish Working-Age Population, 1976–2010

**DOI:** 10.1371/journal.pone.0112314

**Published:** 2014-11-07

**Authors:** Anne Kouvonen, Aki Koskinen, Pekka Varje, Lauri Kokkinen, Roberto De Vogli, Ari Väänänen

**Affiliations:** 1 Department of Social Research, University of Helsinki, Helsinki, Finland; 2 University of Social Sciences and Humanities, Faculty in Wroclaw, Wroclaw, Poland; 3 UKCRC Centre of Excellence for Public Health (Northern Ireland), Queen's University Belfast, Belfast, United Kingdom; 4 Finnish Institute of Occupational Health, Helsinki and Tampere, Finland; 5 Department of Philosophy, History, Culture and Art Studies, University of Helsinki, Helsinki, Finland; 6 Department of Public Health Sciences, School of Medicine, University of California Davis, Davis, United States of America; 7 Department of Epidemiology and Public Health, University College London, London, United Kingdom; Stanford University School of Medicine, United States of America

## Abstract

**Background:**

The health transition theory argues that societal changes produce proportional changes in causes of disability and death. The aim of this study was to identify long-term changes in main causes of hospitalization in working-age population within a nation that has experienced considerable societal change.

**Methodology:**

National trends in all-cause hospitalization and hospitalizations for the five main diagnostic categories were investigated in the data obtained from the Finnish Hospital Discharge Register. The seven-cohort sample covered the period from 1976 to 2010 and consisted of 3,769,356 randomly selected Finnish residents, each cohort representing 25% sample of population aged 18 to 64 years.

**Principal Findings:**

Over the period of 35 years, the risk of hospitalization for cardiovascular diseases and respiratory diseases decreased. Hospitalization for musculoskeletal diseases increased whereas mental and behavioral hospitalizations slightly decreased. The risk of cancer hospitalization decreased marginally in men, whereas in women an upward trend was observed.

**Conclusions/Significance:**

A considerable health transition related to hospitalizations and a shift in the utilization of health care services of working-age men and women took place in Finland between 1976 and 2010.

## Introduction

Change in population-wide disease patterns is a major public health issue. Knowledge of the disease trends and changing burden of disease is essential for estimating the impact of primary prevention, defining public health priorities, and predicting future health care needs [1]. Information on sex-specific patterns is important as different strategies may be needed for women and men. Previous studies examining sex-specific trends in hospitalization have typically focused on specific diseases or diagnostic categories, including diabetes [2], stroke [3], heart failure [4], [5], myocardial infarction [6]–[8], acute coronary syndromes [9], coronary heart disease [10], peripheral artery disease [11], asthma [12], chronic obstructive pulmonary disease [13], and carpal tunnel syndrome [14]. However, research on sex-specific national trends in all-cause hospitalization and hospitalization for main diagnostic categories is rare and information on long-term trends is lacking. In Germany, Nowossadeck [15] analyzed changing rates of hospitalization for individual diagnoses between 2000 and 2009 and found increased rates for congestive heart failure and diseases for spine and back; whereas the hospitalization rates for ischemic heart disease, cerebrovascular diseases and certain cancers decreased. The underlying trends were mainly similar for women and men. However, there were also notable differences; lung cancer hospitalizations decreased in men but sharply increased in women [15].

The individual research findings on shifts in the disease burden based on long follow-ups can be interpreted from the point of view of the health transition theory. This theory suggests that economic, social, cultural and political changes are likely to produce proportional changes in causes of disability and death [16], [17]. It is surprising that – to the best of our best knowledge – there are no previous studies on national hospitalization trends examining long-term proportional changes in disease burden across main diagnostic groups. The present study was set out to investigate the health transition and changing burden of disease in working-age population in Finland; a country which experienced dramatic societal change between the mid-1970s and 2010. In the working-age population the societal shift was reflected in changes in the occupational structure (from agricultural and industrial occupations to service and knowledge-sector occupations) and job content (from physically demanding and chemically hazardous jobs to mentally, socially and cognitively intensive tasks), overall growth of highly-skilled work force, the emergence of late modern work organisations based on the use of information technologies and mobile networks, and considerable changes in leisure time activities [18], [19]. As an indicator of severe health problems and the use of medical treatment we analysed the transition in all-cause hospitalizations and hospitalizations for the main diagnostic categories from 1976 to 2010 in seven representative cohorts of working-age men and women. Hospitalization was measured by the number of discharges rather than by the total number of inpatient days.

## Materials and Methods

### Study population

The total study period was divided into seven five-year time periods (1976–1980, 1981–1985, 1986–1990, 1991–1995, 1996–2000, 2001–2005, and 2006–2010). These seven independent cohorts were constructed by randomly selecting 25% of the 18 to 64-year-old Finns. In total, the study population included 3,769,356 working-age adults (52% men). Data on age and sex were obtained from a population database maintained by Statistics Finland in which every Finnish resident is registered. The dates of death, where applicable, were obtained from the National Death Register kept by Statistics Finland.

### Hospitalization data

Hospitalization data were obtained from the Finnish Hospital Discharge Register that is maintained by the National Institute for Health and Welfare. The data consist of information on all cases of inpatient medical treatment in Finnish public sector hospitals. A recent systematic review showed that completeness and accuracy in this register varies from satisfactory to very good [20]. For each individual the diagnosis data were linked to Statistics Finland records by using national identification numbers. Outcomes were all-cause hospitalization and hospitalizations for the five main diagnostic categories: Diseases of the circulatory system (International Classification of Diseases, Eighth (ICD-8) and Ninth Revision (ICD-9) codes 390–459, International Classification of Diseases, Tenth Revision (ICD-10 codes I00–I99); Mental and behavioral disorders (ICD-8 and ICD-9 codes 291–319; ICD-10 codes F04–F99); Diseases of the musculoskeletal system and connective tissue (ICD-8 and ICD-9 codes 710–739; ICD-10 codes M00–M99); Diseases of the respiratory systems (ICD-8 and ICD-9 codes 460–519; ICD-10 codes J00–J99); and Neoplasms (ICD-8 and ICD-9 codes 140–208; ICD-10 codes C00–C97)). Since the vast majority of pregnancy-related hospitalizations relate to healthy deliveries and therefore do not indicate morbidity, we excluded pregnancy-related causes (ICD8-9 630–679 and ICD10 000–099) from the analyses.

The proportions of the hospitalizations for the five main categories of all first hospitalizations were as follows: musculoskeletal disorders 20%, cardiovascular diseases 15%, respiratory diseases 10%, mental and behavioral disorders 6%, and cancer 4%.

We followed-up the hospitalization data over a five year period in each of the seven cohorts. The follow-up began on 1st January at the beginning of each cohort and ended on the day the participant was hospitalized or died. For the rest of the participants, the follow-up period ended five years after it began, on 31st December.

All records/information was anonymized and de-identified prior to linkage and analysis. Ethical approval for the study was received from the Finnish Institute of Occupational Health.

### Statistical analyses

Age-standardized incidence rates for all-cause and the five main diagnostic categories of hospitalization were calculated separately for men and women and are expressed as the annual number of cases per 10,000 persons in each five-year cohort. For every individual within each cohort, only the first hospitalization for the given diagnostic category was included and the individual was then removed from the risk population (as in the case of death). We did not exclude individuals' subsequent hospitalizations for other diagnostic categories. To detect time trends we applied Cox regression model, which produced age-adjusted sex-stratified proportional hazard ratios (HR) with 95% confidence intervals (95% CI) for all-cause hospitalization and hospitalization for the five main diagnostic categories for each five-year cohort between 1981 and 2010 in relation to the earliest cohort (1976–1980). Due to the random selection method the same individual could appear in several cohorts, but such cases could not be identified and in the analyses the cohorts were assumed to be independent.

All analyses were performed using the SAS 9.2 (SAS Institute, Cary, NC, USA) software.

## Results

Altogether 1,453,190 hospitalizations in men and 1,918,936 hospitalizations in women were recorded in the study cohorts between 1976 and 2010. The mean follow-up time per cohort was 4.97 years.

**Figure 1:**
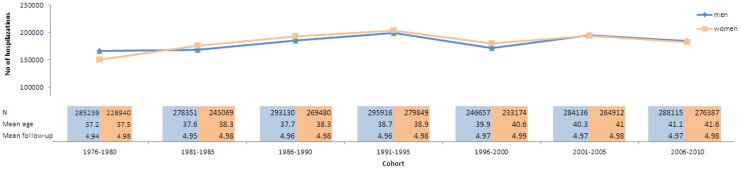
The total number of hospitalizations by cohort in men and women and the mean age and the mean follow-up time in each cohort, Finland 1976–2010.

**Figure 2:**
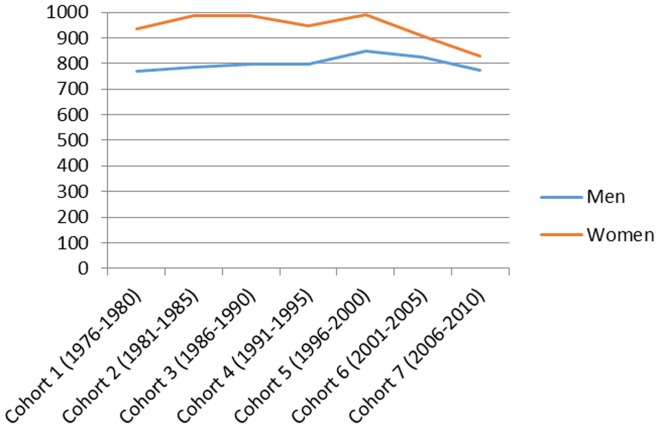
Age-standardized incidence rates per 10,000 individuals for all-cause hospitalizations in men and women, Finland 1976–2010.

**Figure 3:**
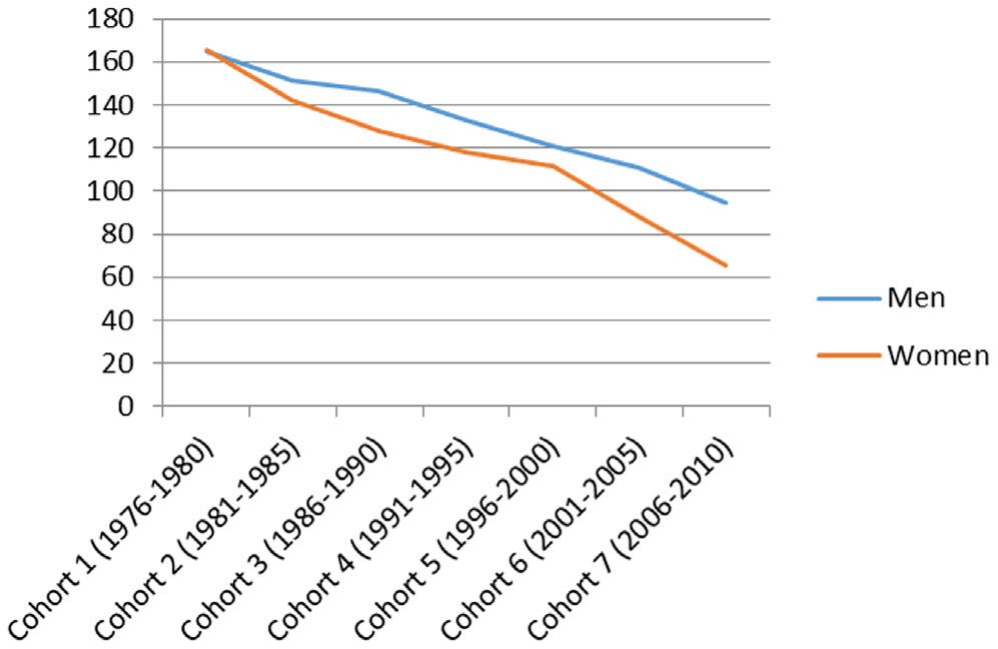
Age-standardized incidence rates per 10,000 individuals for hospitalizations for cardiovascular disease in men and women, Finland 1976–2010.

**Figure 4:**
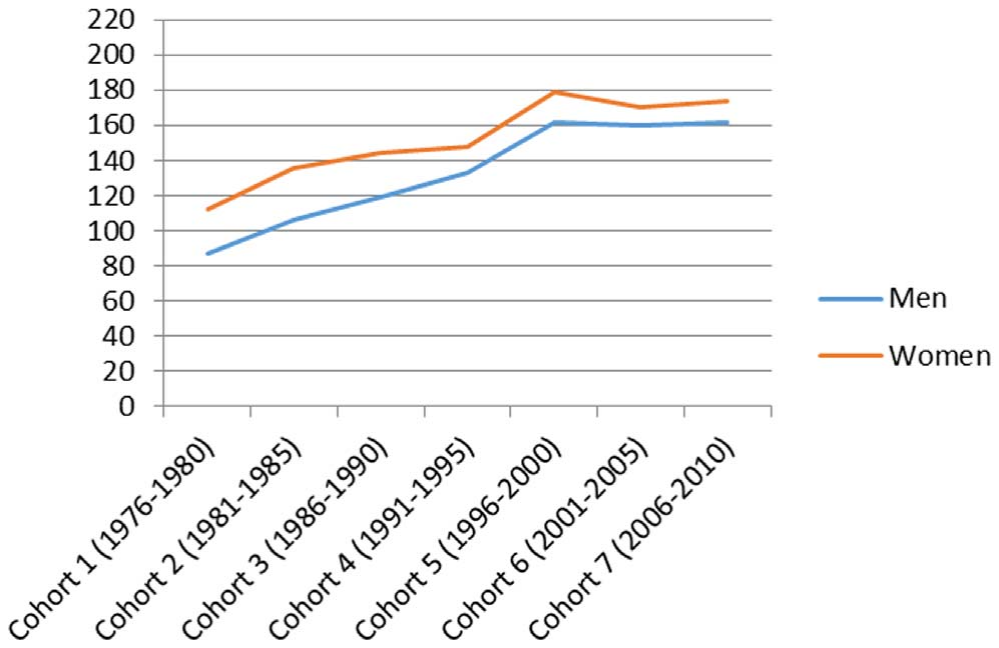
Age-standardized incidence rates per 10,000 individuals for hospitalizations for musculoskeletal disorders in men and women, Finland 1976–2010.

**Figure 5:**
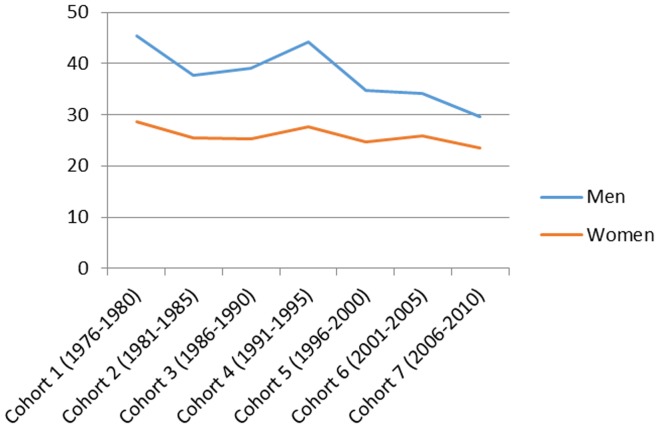
Age-standardized incidence rates per 10,000 individuals for hospitalizations for mental and behavioral disorders in men and women, Finland 1976–2010.

**Figure 6:**
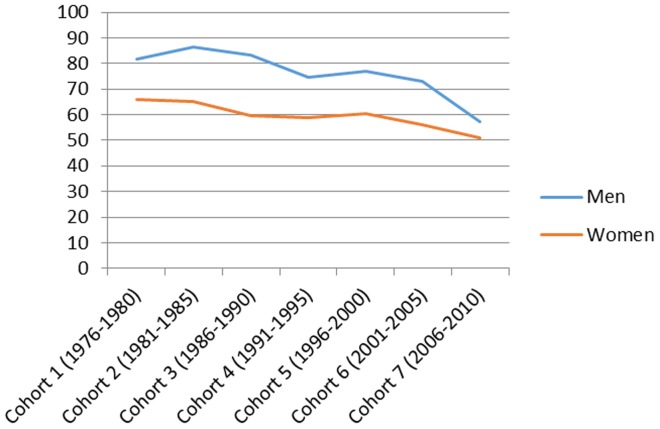
Age-standardized incidence rates per 10,000 individuals for hospitalizations for respiratory diseases in men and women, Finland 1976–2010.

**Figure 7:**
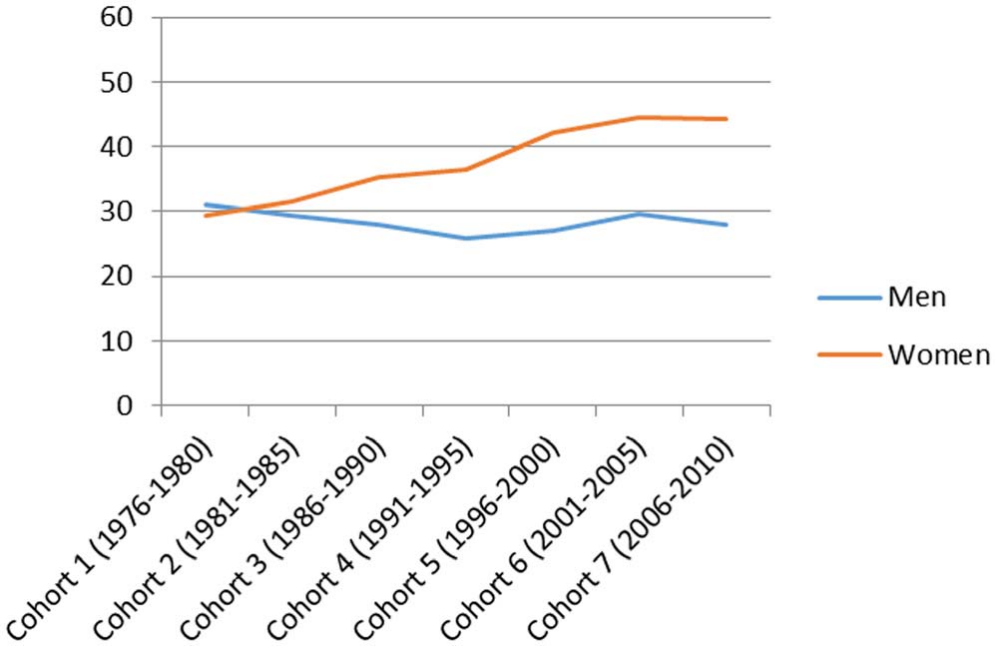
Age-standardized incidence rates per 10,000 individuals for hospitalizations for cancers in men and women, Finland 1976–2010.

Figure 1 presents the total number of hospitalizations by cohort in men and women and the mean age and the mean follow-up time in each cohort. Figures 2–7 present the age-standardized incidence rates per 10,000 individuals for all-cause hospitalization and the five main diagnostic categories in the seven cohorts. Compared to women, men had higher age-standardized rates of hospitalization for cardiovascular diseases and mental and behavioral disorders, whereas age-standardized incidence rates of musculoskeletal disorders and cancer were higher in women. Up until 2001–2005 men had considerably higher levels of hospitalization for respiratory diseases, but the gap has been narrowing and in 2006–2010 the difference was very small.

In men, the age-standardized incidence rate decreased in all main causes of hospitalization apart from musculoskeletal disorders where it rose from 87 in 1976–1980 to 161 in 1996–2000 and remained stable until 2006–2010 (overall increase 85%). In women the age-standardized incidence rate of hospitalizations for musculoskeletal diagnoses increased from 112 in 1976–2010 to 174 in 2006–2010 (overall increase 55%). During the same period, the incidence rate of hospitalization for cardiovascular diseases decreased from 165 to 95 (overall decrease 42%) in men and from 165 to 66 (60%) in women. In a similar way, the incidence rate for respiratory diseases decreased, from 82 to 57 (30%) in men and from 66 to 51 (23%) in women. The incidence rate of hospitalizations for mental and behavioral disorders decreased from 45 to 30 (50%) in men and from 29 to 23 (21%) in women. In men the incidence rate for cancer-related hospitalizations decreased slightly from 31 to 28 (10%), whereas in women it increased from 29 to 44 in (52%). In men, cardiovascular diseases was the most common diagnostic category at the start of the study period, but was replaced by musculoskeletal disorders in 1996–2000. In women, musculoskeletal diagnoses overtook cardiovascular diagnoses as the largest diagnostic category already in 1986–1990.

Table 1 presents sex-stratified age-adjusted proportional risks of all-cause hospitalization and hospitalizations for the five main diagnostic categories among men and women in 1981–2010 using the first cohort (1976–1980) as the reference group. With the exception of cancer, the time trends were very similar in men and women. The risk of all-cause hospitalization remained relatively stable throughout the study period. The risk of hospitalization for cardiovascular diseases decreased systematically throughout the study period (HR 0.57; 95% CI: 0.56, 0.58 in 2006–2010 in men and HR 0.39; 95% CI: 0.38, 0.40 in 2006–2010 in women). The risk of hospitalization for musculoskeletal disorders increased, but the increase levelled off already in 1996–2000 (HR 1.83; 95% CI: 1.79, 1.88 in 2006–2010 in men and HR 1.52; 95% CI: 1.48, 1.55 in 2006–2010 women). The risk of hospitalization for mental and behavioral disorders decreased slightly (HR 0.63; 95% CI: 0.61, 0.66 in 2006–2010 in men and HR 0.81; 95% CI: 0.78, 0.85 in 2006–2010 in women).

**Table 1:** Age-adjusted Proportional Hazard Ratios (HR) and their 95% Confidence Intervals for Hospitalization in Relation to the First Cohort in Seven Consecutive Cohorts, Finland, 1976–2010.

	*Men*			*Women*		
*All-cause*	*N*/Cases	HR	95% CI	*N*/Cases	HR	95% CI
1976–1980	285,239/81,164	1.00	reference	228,940/80,894	1.00	reference
1981–1985	278,351/80,108	1.03	1.02, 1.04	245,069/89,577	1.04	1.03, 1.05
1986–1990	293,130/86,562	1.06	1.05, 1.07	269,480/98,681	1.05	1.04, 1.06
1991–1995	295,916/88,868	1.05	1.04, 1.04	279,849/101,007	1.00	0.99, 1.01
1996–2000	246,657/79,058	1.13	1.12, 1.14	233,174/88,648	1.04	1.03, 1.05
2001–2005	284,136/90,563	1.10	1.09, 1.11	264,912/93,473	0.94	0.93, 0.95
2006–2010	288,115/88,795	1.02	1.01, 1.03	276,387/91,591	0.87	0.86, 0.88
	Cases	HR	95% CI	Cases	HR	95% CI
*Cardiovascular diseases*						
1976–1980	16,670	1.00	reference	15,500	1.00	reference
1981–1985	15,024	0.92	0.90, 0.94	14,505	0.85	0.83, 0.87
1986–1990	15,298	0.89	0.87, 0.91	14,256	0.76	0.74, 0.77
1991–1995	14,836	0.79	0.78, 0.81	14,194	0.70	0.68, 0.71
1996–2000	11,990	0.73	0.71, 0.74	11,903	0.66	0.64, 0.67
2001–2005	13,124	0.67	0.65, 0.74	10,775	0.52	0.50, 0.53
2006–2010	12,441	0.57	0.56, 0.58	8797	0.39	0.38, 0.40
*Musculoskeletal disorders*						
1976–1980	10,900	1.00	reference	10,965	1.00	reference
1981–1985	13,097	1.23	1.20, 1.26	14,230	1.20	1.17, 1.23
1986–1990	15,566	1.39	1.36, 1.42	16,631	1.28	1.25, 1.31
1991–1995	17,975	1.54	1.50, 1.57	18,470	1.31	1.28, 1.34
1996–2000	18,524	1.86	1.81, 1.90	19,416	1.57	1.53, 1.61
2001–2005	20,987	1.82	1.78, 1.86	20,953	1.48	1.45, 1.51
2006–2010	21,744	1.83	1.79, 1.88	22,673	1.52	1.48, 1.55
*Mental and behavioral disorders*						
1976–1980	6624	1.00	reference	3291	1.00	reference
1981–1985	5392	0.83	0.80, 0.86	3132	0.89	0.84, 0.93
1986–1990	5873	0.86	0.83, 0.89	3387	0.87	0.83, 0.91
1991–1995	6574	0.95	0.92, 0.98	3938	0.96	0.92, 1.01
1996–2000	4334	0.74	0.72, 0.95	2970	0.86	0.82, 0.91
2001–2005	4877	0.73	0.70, 0.76	3489	0.90	0.86, 0.94
2006–2010	4226	0.63	0.61, 0.66	3229	0.81	0.78, 0.85
*Respiratory diseases*						
1976–1980	11,326	1.00	reference	7449	1.00	reference
1981–1985	11,122	1.07	1.04, 1.09	7603	0.91	0.89, 0.94
1986–1990	11,343	1.04	1.01, 1.07	7611	0.96	0.93, 0.99
1991–1995	10,349	0.95	0.92, 0.97	8009	0.87	0.84, 0.90
1996–2000	8557	0.98	0.95, 1.01	6835	0.91	0.88, 0.94
2001–2005	9835	0.93	0.91, 0.96	7220	0.84	0.81, 0.87
2006–2010	7991	0.73	0.71, 0.75	6893	0.76	0.74, 0.79
*Cancers*						
1976–1980	3002	1.00	reference	2589	1.00	reference
1981–1985	2726	0.94	0.89, 0.99	3088	1.08	1.03, 1.14
1986–1990	2736	0.91	0.87, 0.96	3715	1.21	1.15, 1.27
1991–1995	2777	0.85	0.81, 0.89	4290	1.26	1.20, 1.33
1996–2000	2471	0.87	0.83, 0.92	4439	1.45	1.38, 1.52
2001–2005	3361	0.97	0.92, 1.02	5421	1.52	1.45, 1.59
2006–2010	3666	0.90	0.66, 0.95	5987	1.51	1.45, 1.59

The risk of hospitalization for respiratory diseases has declined both in men and women. The risk of cancer hospitalization has decreased very slightly in men, whereas in women a constant upward trend in the risk can be observed (HR 1.51; 95% CI: 1.45, 1.59 in 2006–2010).

## Discussion

The present study examined incidence rates and proportional risks for all-cause hospitalization and for the five main diagnostic categories in seven representative consecutive cohorts of Finnish working-age men and women between 1976 and 2010. The findings show that musculoskeletal disorders have increased their proportion of the total of all five diagnostic categories that were included in this study whilst the proportion of cardiovascular diseases has decreased. The observed overall trends were very similar in men and women with the exception of cancer: these hospitalizations marginally decreased in men but increased in women. The findings provide new evidence which specifies the health transition theory. It seems that the general transition occurs in hospitalizations when the nation moves towards the late modern societal order but the pattern of the changes is partly different for women and men. The general secular shifts and sex-specific findings are probably related to notable changes in lifestyles and in living and working conditions but at the same time they also are likely to be connected to developments in health care, health promotion, and health policies.

### Cardiovascular disease

Our findings demonstrate a declining incidence of hospitalization for cardiovascular diseases; the decrease being a slightly larger in women. Studies from other national samples show that age-adjusted coronary heart disease and cerebrovascular disease hospitalization rates have significantly decreased [21]. The role of widespread primary prevention and decreases in conventional risk factors have been highlighted as important contributors to these reductions; including decreases in smoking prevalence and population wide levels of systolic blood pressure as well as favorable lipid effects [22], [23]. The widespread and increasing use of antihypertensive drugs as well as statins and other lipid-lowering drugs, has probably also contributed to these trends [24], [25]. However, it has been predicted that increasing prevalence of overweight and obesity may be compromising favorable trends in the risk factors in the future [23].

Moreover, it is well known that trends in cardiovascular diseases may be set decades before the conditions become manifest [26], and that early-life poor socio-economic conditions are associated with a higher cardiovascular risk later in life [27]; an important part of this is their potential effect on development of conventional risk factors [28]. It is therefore plausible that population-wide improvements in childhood socio-economic environments have contributed to declining cardiovascular risk. In addition, decreases in cardiovascular risk factors relate to an upward shift in educational attainment; a substantially smaller proportion of the population than before is exposed to the risk associated with low education [28]. The contribution of educational shift has been shown to be somewhat stronger for women than for men. In addition, changes in policies, such as introduction of comprehensive smoke-free policy, may have helped to change social norms [29].

### Musculoskeletal disorders

Our results show a clear increase in hospitalizations for musculoskeletal disorders. Indeed, musculoskeletal disorders have become the most common reason for claiming sickness allowance in Finland [30]. Hospitalization due to musculoskeletal disorders has also increased in Germany [15]. In the US health care utilisation for spine conditions, the prevalence of chronic impairing low back pain, and back and neck-related health expenditure have increased [31], [32]. In Finland it has been noted that there have been improvements in diagnostic and therapeutic methods that reveal an increasing range of musculoskeletal disorders [33].

Increasing rates of musculoskeletal hospitalization may be attributable to changes in the nature of working life: the shift from manual types of jobs towards jobs involving more sedentary tasks such as more office-based and computer-based jobs. It is now acknowledged that for a large number of employees, lack of physical activity – both at work and on leisure time - is a major risk factor for musculoskeletal ill health [34]. At the same time, obesity, which is a risk factor for particularly certain musculoskeletal conditions [35], has rapidly increased.

Work-related psychosocial stress has also been linked to hospitalizations for musculoskeletal disorders [36]. Because of the globalisation of work, widespread information technology and a growing pressure to increase productivity and more stressful psychosocial environments and working patterns are now affecting increasing proportions of both the male and female workforce [37].

### Mental and behavioral disorders

The risk of psychiatric hospitalization decreased slightly. Mental disorders as a reason for sickness absence and disability pension have increased in recent decades in Finland [33]. As in most high income countries, mental health service in Finland has been dramatically transformed by an increase in ambulatory treatment and care. However, despite decrease in the total number of hospital beds, the annual number of psychiatric in-patients has remained largely unchanged over time; this is due to the decrease in average length of psychiatric hospital stay [38].

### Respiratory diseases

Our data show that the risk of hospitalization for respiratory diseases has decreased. In a similar way, in Danish working population, the number of hospitalizations for chronic lower respiratory diseases has been reduced over time [39].

The decrease in hospitalizations both for cardiovascular and respiratory diseases can be partially explained by widespread smoke-free legislation, introduced in Finland from 1990s onwards. A recent meta-analysis showed that smoke-free legislation is associated for a lower risk of hospitalization for cardiac, cerebrovascular and respiratory diseases [40].

In addition, considerable regulatory measures have been taken to prevent the adverse health outcomes of hazardous substances, for example all asbestos usage has been banned and lead-exposed workers are being biologically monitored [41]. Occupational inhalation exposure to most chemical agents has decreased in Finland since 1970s and chemical exposures and related disease burden are expected to further decrease in the future [42].

### Cancer

Our results showed that the risk of cancer hospitalization decreased marginally in men, whereas in women an upward trend in risk can be observed. An earlier study in the Finnish general population similarly showed that in men there have been only minor changes in the age-standardized total cancer incidence rates, whereas in women the total cancer morbidity has slightly increased since the 1950s [43].

A number of reasons may have contributed to increasing cancer rates in working-age women. First, the risk of breast cancer has continuously increased. The introduction of organised mammographic screening programme affects the breast cancer incidence rate in a population as the diagnosis is advanced in time [44]. In Finland national mammographic screening was introduced in 1987; in that year the incidence of breast cancer increased by about one-tenth [45]. An analysis of secular trends which corrected for the influence of screening showed that in Finland the breast cancer rates increased by 13% per 5-year period [44]. Another important contributing factor for the increased breast cancer risk relates to the increased use of postmenopausal hormone therapy (HT) [45]. A study examining the HT use and breast cancer risk in Nordic countries in 1995 showed that in three other Nordic countries (Sweden, Norway and Iceland) that had had a significant drop in HT use, the increasing trends of breast cancer incidences either experienced a down-turn or the increase declined whereas in Finland, with only a small decline in HT, this was less evident [46].

Second, strong and consistent increases in endometrial cancer have been reported over the last 50 years; the biggest increase has been seen in postmenopausal women but in the last decade the rates have also increased in younger women [47].

Third, in Finland smoking has systematically decreased in men already over several decades whereas in women smoking has started to decline only in recent years. As a consequence, the incidence of lung cancer has constantly increased in women and is projected to continue to increase [45].

Finally, obesity has strongly increased over the last three decades and it is well established that excess body adiposity is a risk factor for cancer development [48]. It has also been shown that both in relative and absolute terms, obesity–related cancer is a greater problem for women than men; and endometrial, breast and colorectal cancers have been identified as priorities for research and public health measures [48].

### Strengths and limitations

A major strength of our study was that the hospitalization data were derived from a national hospital discharge register with proven good accuracy and coverage [20]. There was no loss to follow-up and the changes in International Classification of Diseases were recoded to correspond to the most recent classification. As far as we are aware, this was the first study to examine long-term national trends in main causes of hospitalization among working-age men and women.

Despite these strengths, it is also important to acknowledge potential limitations. First, we used hospitalization as a proxy for underlying morbidity. Hospitalization is considered to be a reliable indicator of the use of medical treatment [49]. However, the long-term shifts in the use of hospital services and proportional share of diagnoses related to hospitalizations within a population are also likely to reflect changes in treatments and options of health care. Moreover, over time changes in coding practices and diagnostic criteria may have affected hospitalization trends for some causes and proportional shares of main diagnostic categories.

Second, referral bias and differential access to hospital services might have affected the results [50]. Self-referral and referral by general practitioners may be influenced by a number of factors such as geography, employment status, age, education, and economic status [50]. There are significant differences in health care service provision between local authorities and significant socioeconomic and regional inequities in the provision of and access to health care services in Finland [33], [51].

Third, we considered only the first hospitalization for any of the five diagnostic categories and excluded any subsequent hospitalizations for the same category.

## Conclusions

The present study demonstrates that there have been significant secular shifts in main causes of hospitalization and consequently the content of disease burden requiring hospital treatment has considerably changed among the Finnish working-age population between 1976 and 2010. This study provides new evidence of the health transition in a nation that has moved towards the late modern spheres of life within a short period of time. Generally the main trend both in men and women was away from cardiovascular and respiratory diseases and towards musculoskeletal disorders. Interestingly, cancer treatments in hospitals decreased marginally in men but clearly increased in women. The exact reasons for the observed trends are not known. However, it is likely that two types of factors – health interventions and socio-economic and lifestyle changes - have affected these trends. Health interventions include better control of many cardiovascular risk factors and advances in medical treatments. Socio-economic and lifestyle changes beyond the health sector include changes in working life and in the occupational distribution of the labor force, higher educational levels, increased social mobility, increased use of digital technology, more sedentary work and lifestyles, and increasing obesity rates. The present findings can have significant policy implications. Awareness of sex-specific long-term temporal changes in main causes of hospitalizations can help to identify public health priorities and guide health care planning.
